# Polymer Nanocomposite Sensors with Improved Piezoelectric Properties through Additive Manufacturing

**DOI:** 10.3390/s24092694

**Published:** 2024-04-24

**Authors:** Rishikesh Srinivasaraghavan Govindarajan, Zefu Ren, Isabel Melendez, Sandra K. S. Boetcher, Foram Madiyar, Daewon Kim

**Affiliations:** 1 Department of Aerospace Engineering, Embry-Riddle Aeronautical University, Daytona Beach, FL 32114, USA; srinivr1@my.erau.edu (R.S.G.); renz@my.erau.edu (Z.R.); 2 Department of Mechanical Engineering, Embry-Riddle Aeronautical University, Daytona Beach, FL 32114, USA; melendei@my.erau.edu (I.M.); sandra.boetcher@erau.edu (S.K.S.B.); 3 Department of Physical Science, Embry-Riddle Aeronautical University, Daytona Beach, FL 32114, USA

**Keywords:** additive manufacturing, piezoelectric, polymer, nanocomposite, BNNTs

## Abstract

Additive manufacturing (AM) technology has recently seen increased utilization due to its versatility in using functional materials, offering a new pathway for next-generation conformal electronics in the smart sensor field. However, the limited availability of polymer-based ultraviolet (UV)-curable materials with enhanced piezoelectric properties necessitates the development of a tailorable process suitable for 3D printing. This paper investigates the structural, thermal, rheological, mechanical, and piezoelectric properties of a newly developed sensor resin material. The polymer resin is based on polyvinylidene fluoride (PVDF) as a matrix, mixed with constituents enabling UV curability, and boron nitride nanotubes (BNNTs) are added to form a nanocomposite resin. The results demonstrate the successful micro-scale printability of the developed polymer and nanocomposite resins using a liquid crystal display (LCD)-based 3D printer. Additionally, incorporating BNNTs into the polymer matrix enhanced the piezoelectric properties, with an increase in the voltage response by up to 50.13%. This work provides new insights for the development of 3D printable flexible sensor devices and energy harvesting systems.

## 1. Introduction

The rapid evolution of microelectronics technology has catalyzed significant breakthroughs in various scientific fields, particularly in the realm of smart sensor technology. These advancements offer unprecedented versatility, paving the way for the development of next-generation conformal electronics across diverse applications. Traditional micro-scale electronic fabrication techniques, such as imprint lithography, micromachining, and photolithography, have primarily relied on two-dimensional (2D) rigid substrates [1,2,3,4]. However, these methods face inherent limitations in achieving non-planar structures for complex curvilinear architectures. Recent advances in materials, manufacturing techniques, and microelectromechanical (MEMS) designs have made a substantial contribution to the emergence of several smart devices based on piezoelectric materials for society [5,6,7]. Piezoelectric materials, a family of organic or inorganic materials, are renowned for their ability to convert mechanical stress into electrical charge (direct effect) or vice versa (inverse effect), thus playing a pivotal role in a wide range of multidisciplinary areas, including the aerospace and bio-medical fields [8,9], to detect and sense physical phenomena such as mechanical strains and pressure [10,11,12,13]. 

Traditionally, ceramics like lead zirconate titanate (PZT), barium titanate (BaTiO_3_), and calcium copper titanate (CCTO) have been preferred due to their exceptional piezoelectric characteristics [14,15,16]. Conversely, their rigidity, brittleness, toxicity, and high density limit their application in conformal electronics. To circumvent these challenges, researchers have developed polymer-based composites, capitalizing on polymers’ unique properties, such as mechanical flexibility, lightweightness, and ease of processing. Particularly, polyvinylidene fluoride (PVDF), a fluoropolymer, with its distinct polymorphism (*α*, *β*, *γ*, and *δ* phases), has emerged as a leading candidate owing to its high dielectric constant compared to other polymers, increased flexibility, and long-term stability under high electric fields [17,18]. The performance of PVDF-based sensors is enhanced by incorporating micro and nano piezoelectric fillers into the polymer matrix, which synergize the flexibility and high piezoelectric property, thereby unlocking new functionalities tailored to envisioned applications [19]. Traditional processes, such as compression molding, spin coating, and solvent casting, have been employed for manufacturing piezocomposite materials [11,20,21]. However, these traditional methods are time-consuming, limit design flexibility, and are mostly suitable for large-scale structures with complex fabrication processes, underscoring the need for alternative approaches.

In contrast, additive manufacturing (AM) technology presents a promising solution with its layer-by-layer stacking approach, enabling the creation of intricate three-dimensional (3D) structures with unparalleled ease and efficiency. This capability holds immense potential for the fabrication of multifunctional materials with complex geometries, thereby revolutionizing sensor, energy harvester, and actuator devices [22,23,24]. Although attempts have been made to 3D print PVDF-based sensing devices using micro-dispensing [25,26] and fused deposition techniques [27,28], challenges persist in printing complex out-of-plane patterns, producing uniform filaments and manufacturing an array of sensor materials in a time-efficient manner. Among different AM techniques, ultraviolet (UV)-based 3D printing overcomes the aforementioned fabrication challenges. Stereolithography (SLA), a well-known UV-based 3D printing technique, can produce high-quality, macro-sized 3D structures. Yet, this method uses a laser beam that possesses a low printing rate and is not suitable for rapid production [29]. In this study, a liquid crystal display (LCD)-based printing method is employed as this is economical and can print an entire layer at once with effective resolution. Despite advancements in printing technology, to the author’s knowledge, the availability of resin material amenable to UV-based 3D printing processes remains a pressing challenge, highlighting the need for developing an optimized material exhibiting flexibility and piezoelectric properties. 

On the other hand, the selection of piezocomposite materials plays a vital role in material synthesis in terms of compatibility, lightweightness, and piezoelectric properties. Boron nitride nanotubes (BNNTs), a promising nanofiller, are known for their high thermal and chemical stability, mechanical strength, and good biocompatibility. This nanofiller offers the potential to significantly enhance the piezoelectric properties of polymer composites, making them ideal for highly sensitive force-sensing applications [30,31,32]. Although BNNTs’ piezoelectric nature has been explored through analytical and simulation studies [33,34,35], experimental research on incorporating BNNTs into polymer composites, especially for 3D printing smart sensors, remains in the nascent stage. 

Addressing the imperative requirements, this paper presents a comprehensive investigation into the structural, thermal, rheological, mechanical, and piezoelectric properties of a newly developed sensor resin material based on PVDF. Additionally, the effect of incorporating BNNTs as a nanofiller is examined, focusing on enhancing the nanocomposite’s piezoelectric sensor response. Leveraging an LCD-based 3D printer, this study also investigates the micro-scale printability of the developed sensor material.

## 2. Materials and Methods

### 2.1. Sample Preparation

The process of developing a polymer resin involves blending the base polymer with additives that enable UV curability, suitable for the selected 3D printing process. The primary component of the developed resin was PVDF, a base polymer (Sigma Aldrich, St. Louis, MO, USA) with an average particle size of 3–10 µm, molecular weight (Mw) of ~534,000 g/mol, and density of 1.74 g/mL. Additionally, a hexamethylene glycol diacrylate (HDODA) monomer with a density of 1.01 g/mL, bisacylphosphine oxide (BAPO) photoinitiator (Sigma Aldrich, St. Louis, MO, USA), and Sudan I UV absorber (Thermo Fisher Scientific, Waltham, MA, USA) were uniformly mixed with a diethyl fumarate (DEF) solvent (Sigma Aldrich, St. Louis, MO, USA). The investigation examined samples with a base polymer range of 20 wt. % to 40 wt. % in increments of 5 wt. % while maintaining fixed amounts of UV absorber, photoinitiator, and solvent at 0.1%, 1.9%, and 23% by weight, respectively. The remaining quantity consisted of monomers, ranging between 35% and 55% by weight. Additionally, 2 wt. % of boron nitride nanotubes (BNNTs) with an assay exceeding 80% (Sigma Aldrich, St. Louis, MO, USA) was incorporated as received without further purification to develop a nanocomposite owing to its higher piezoelectricity and fine electric field tunability. To fabricate the piezoelectric resin, PVDF with respective wt. % proportions was mixed using a centrifugal planetary THINKY ARM-310 mixer (Laguna Hills, CA, USA) at 2000 rpm for an average duration of 20 min, ensuring thorough mixing of the selected combinations without any agglomeration or air bubbles. Table 1 presents the sample combinations used throughout various tests. 

### 2.2. Piezoelectric Substrate Development Procedure

**Figure 1:**
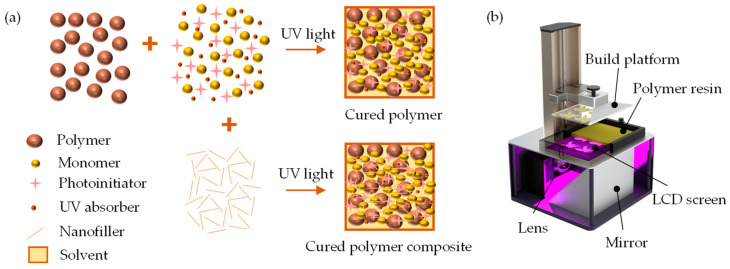
(**a**) Schematic representation for UV curability of polymer dissolved in DEF solvent and (**b**) LCD 3D printing setup.

The piezoelectric-based sensor was fabricated using the Phrozen Sonic Mini 8K LCD 3D printer (Phrozen Technology, Hsinchu City, Taiwan), which boasts a resolution of up to 1152 ppi and exceptional stability while printing, as depicted in Figure 1. To assess the UV curing behavior of the developed piezoelectric resin, an array of square coupons (5 mm × 5 mm × 1 mm) were modeled and sliced using the Phrozen 3D software. 

Given the customized nature of the developed resin, optimal printing parameters were determined through iterative trials with a focus on critical factors, such as delamination between layers, build platform adhesion, and exposure time for polymerization. Table 2 lists the parameters used for printing both the developed polymer and nanocomposite resins. After printing, the coupons underwent a 5 min rinse in DEF and then in isopropanol (IPA) to eliminate any residual uncured resin.

### 2.3. Phase Characterization Methods

Fourier transform infrared spectroscopy (FTIR) transmission spectra of the polymer with added nanofiller were recorded within a wavenumber range of 650–3500 cm^−1^ using an Agilent Cary 630 spectrometer (Santa Clara, CA, USA) with 64 scans per spectrum. X-ray diffraction (XRD) spectra were obtained within a 2θ range from 10° to 50° using a Panalytical X’Pert Pro diffractometer (Malvern, Worcestershire, UK) with a Cu radiation source operating at 45 kV and 40 mA, with an irradiated length of 5 mm.

### 2.4. Thermal and Rheological Measurements

The melting and crystallization properties of the developed polymer and nanocomposite materials were investigated based on differential scanning calorimeter (DSC) measurements collected using a Mettler Toledo DSC 3 (Columbus, OH, USA). Samples weighing between 30 mg and 40 mg were contained in 40 µL aluminum crucibles. The temperature of the samples was gradually increased from 30 °C to 180 °C at a 10 °C/min heating rate. 

The rheological measurements of the mixed resins with varying proportions of polymer and with nanofillers were conducted utilizing a TA Instruments HR 20 hybrid rheometer (New Castle, DE, USA). The testing framework comprised two 25 mm diameter parallel plates and a 0.75 mm gap, with temperature control maintained by a Peltier plate setup. Two types of measurements were performed: (1) viscosity over a varying shear rate from 1 s^−1^ to 100 s^−1^ at 25 °C and (2) viscosity at a constant shear rate of 100 s^−1^ for a 60 s duration at 25 °C.

### 2.5. Nanoindentation

Mechanical characterization of 3D printed samples was conducted using a Bruker Hysitron TI-980 nanoindenter (Billercia, MA, USA) equipped with a 10 mN low-load transducer. To study the material’s reduced modulus and viscoelastic properties, single indentations and micro-scale dynamic mechanical analysis were performed, respectively, with an applied peak force of 5 mN and a 5 s dwell time at 100 Hz frequency.

### 2.6. Piezoelectric and Dielectric Property Measurements

The piezoelectric strain coefficient (*d_33_*), which represents the induced polarization per unit stress applied in the thickness direction, was measured using an APC YE2730A piezometer (Mackeyville, PA, USA) with a 250 mN applied force. The dielectric constant was determined through parallel plate capacitor measurements of 3D printed samples sandwiched between aluminum plates. Capacitance values were obtained with a high-precision Hioki IM 3570 impedance analyzer (Dallas, TX, USA) across a frequency range of 100 Hz to 1 MHz. The dielectric constant (*ε_r_*) was calculated as *ε_r_ = C·d/ε_0_·A*, where *C* represents the measured capacitance at different frequencies, *A* denotes the area of the aluminum electrode, *d* signifies the thickness of the individual 3D printed substrate, and *ε_0_* is the free space dielectric constant. Additionally, the piezoelectric voltage constant (*g_33_*) was derived using the relation *g_33_* = *d_33_/ε_r_ ε_0_* based on the measured values. Finally, the electrical output signal from the 3D printed device was captured using an Agilent DSO-X-4024A digital oscilloscope (Keysight Technologies, Santa Rosa, CA, USA).

## 3. Results and Discussions

In this section, the structural, rheological, and thermal properties of the developed resin are presented. Additionally, the mechanical and piezoelectric properties of the 3D printed sensor material are discussed. Considering better printability and longevity among different polymer combinations, resin with 35 wt. % PVDF was selected for nanofiller addition.

### 3.1. Structural and Phase Characterization

**Figure 2:**
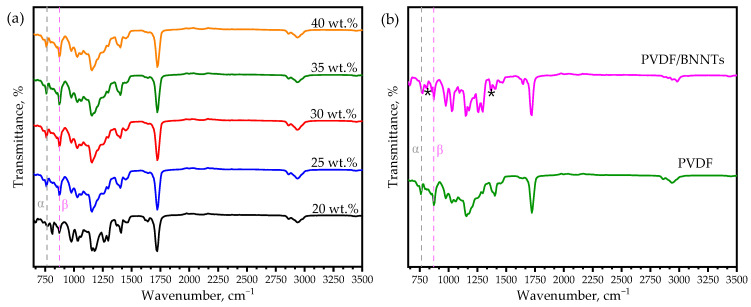
FTIR transmittance spectra: (**a**) resins with different PVDF wt. % and (**b**) BNNT filler addition to PVDF polymer, with unique peaks marked (*) corresponding to B-N bond.

The FTIR spectra analysis of the developed resin with varying polymer content provides information about the crystalline structure, which is crucial for determining the piezoelectric properties. Among different polar and non-polar crystallographic phases of PVDF, the *β* phase is the most essential form due to its excellent piezoelectric behavior. In the polar *β* phase, the polymer chains exhibit an ordered carbon backbone structure with fluorine and hydrogen atoms on each side, enhancing the material’s piezoelectric properties. Transmittance peaks at 762 cm^−1^ (CF_2_ bending and rocking), 872 cm^−1^ (C-F stretching), and 1062 cm^−1^ (C-C-C bonding) represents the non-polar *α*-phase, while peaks at 846 cm^−1^ (CF_2_ stretching and CH_2_ rocking), 1179 cm^−1^ (C-C bonding, monomer’s C-O stretching vibration), 1406 cm^−1^ (CH_2_ wagging, monomer’s C=C), and 1423 cm^−1^ (CH_2_ bending) signify the polar *β* phase presence in the PVDF polymer [25,36,37,38], as shown in Figure 2a. Moreover, peaks at 1636 cm^−1^ (C=C bond), 1719 cm^−1^ (C=O stretching vibration), 2864 cm^−1^ (=C-H stretching vibration), and 2938 cm^−1^ (CH_2_ symmetrical) indicate the presence of a monomer in the developed polymer resin [39]. Overall, an increment in the polar peak at 835 cm^−1^ was noticed with an increase in PVDF polymer content, which was validated further by quantifying the polar phase fraction. Furthermore, spectra of the sample containing BNNTs revealed a unique peak at 810 cm^−1^ and 1361 cm^−1^ corresponding to in-plane and out-of-plane stretching vibrations of B-N bond [40], as shown in Figure 2b.

Based on the Beer–Lambert law, the relative fraction of the *β* phase was quantified as shown in Equation (1):

(1) $$F_{\beta }=\frac{A_{\beta }}{\left(\frac{k_{\alpha }}{k_{\beta }}\right)A_{\alpha }+A_{\beta }}$$

where *A_α_* and *A_β_* are the absorbance peak intensities at the 762 cm^−1^ (*α* phase) and 846 cm^−1^ (*β* phase); *k_α_* and *k_β_* are the absorption coefficients, whose values were 7.7 × 10^4^ cm^2^ mol^−1^ and 6.1 × 10^4^ cm^2^ mol^−1^, respectively. The calculated polar phase fraction for different composition types is listed in Table 3; a maximum of 64.89% polar phase was achieved with nanofiller addition.

**Figure 3:**
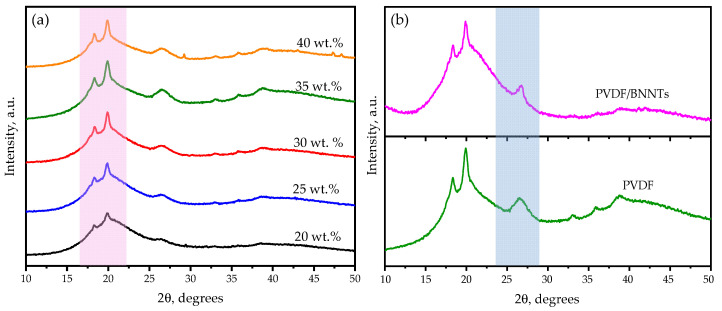
XRD diffraction patterns of (**a**) resins with different PVDF wt. % and (**b**) BNNT filler addition to PVDF polymer.

The XRD diffraction peaks were analyzed to investigate the crystal phases of the polymer and the impact of the nanofiller on overall crystallization, as shown in Figure 3. The diffraction peaks at 2θ = 17.8°, 18.4°, 20.0°, 26.6°, and 38.6°, shown in Figure 3a, were attributed to the diffractions of (100), (020), (110), (021), and (040) crystal planes of PVDF polymer [41,42], respectively, with an increment in intensity corresponding to high polymer content. Peaks near 26.4° and 44.3° in Figure 3b were associated with BN crystal planes, exhibiting a drop in intensity ascribed to the nucleation effect of PVDF crystallization, which was also quantified using thermal analysis [43]. Additionally, an increase in intensity was observed for the 20.5° (110/200) polar phase plane, which supported the higher *β* phase presence with the developed resin [44].

### 3.2. Thermal Analysis of Developed Resins Using DSC

**Figure 4:**
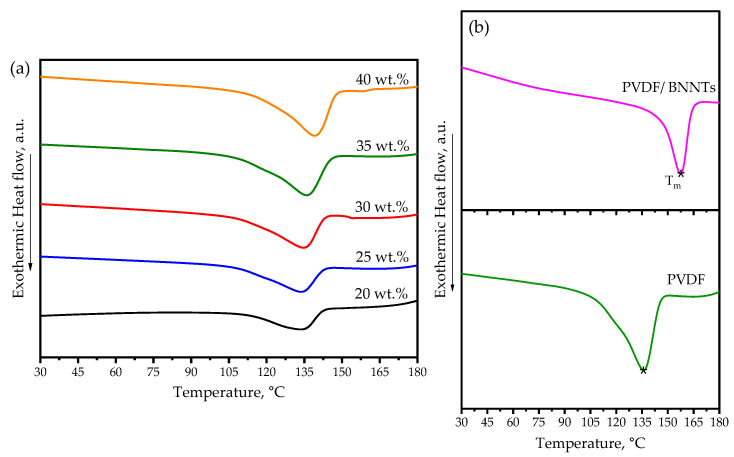
Heat flow curves as a function of temperature: (**a**) different wt. % of PVDF polymers and (**b**) comparison of PVDF with BNNT addition, with melting peak marked (*).

The DSC thermograms depicting samples with an increase in PVDF content and nanofiller addition are displayed in Figure 4a,b. Thermal analysis plays a crucial role in assessing the thermal stability and reliability of sensor materials across a wide temperature range. 

Thermal parameters, including melting temperature (*T_m_*) and melting enthalpy (Δ*H_m_*), are summarized in Table 4, based on the measured thermograms, while the crystallinity content (*X_c_*) was calculated using Equation (2):

(2) $$X_{c}=\frac{∆H_{m}}{∆H_{m}^{*}}\times 100\%$$

where ∆$H_{m}^{*}$ is the measured melting enthalpy corresponding to the pure crystalline PVDF (32.11 J/g).

Overall, the results indicated that as the PVDF content increased, the melting temperature, enthalpy, and crystallinity increased. The observed rise in *T_m_*, Δ*H_m_*, and *X_c_* can be attributed to an increase in the crystalline phase alongside PVDF content. The crystalline phase typically requires a significant amount of energy to change phase when compared to an amorphous phase. Notably, an increase of 5.35 °C in *T_m_* and 5.99 J/g in Δ*H_m_* was observed when the maximum and minimum amount of polymer wt. % were investigated in this study. Upon the introduction of BNNT nanofillers, as shown in Figure 4b, the *T_m_* exhibited a significant increase compared to the PVDF polymer. The presence of BNNTs affected the Δ*H_m_* by creating a nucleation site, thus providing an insufficient area for crystal formation and alignment. Finally, it is essential to note that the thermogram profiles were single-peaked, an indicative of a homogenous composite that melted uniformly during the heating process.

### 3.3. Rheological Behavior of Developed Resins

For the custom-developed resins with different polymer contents, the investigation of rheological behavior plays a crucial role in the selected 3D printing process. Understanding the viscosity of combinations with different PVDF wt. % contents under conditions that emulate the shear rate of LCD printing is of paramount importance. Unlike extrusion-based AM techniques that involve high shear forces, vat-based processes like LCD printing result in significantly lower shear forces as the printed material remains relatively static on the building platform, which is displaced at a low speed to prevent agitation that may affect the curing process and accuracy of the printed component. However, despite being categorized as a low-shear manufacturing process, LCD 3D printing still involves movement and flow within the vat, influenced by the rheological behavior of the material.

**Figure 5:**
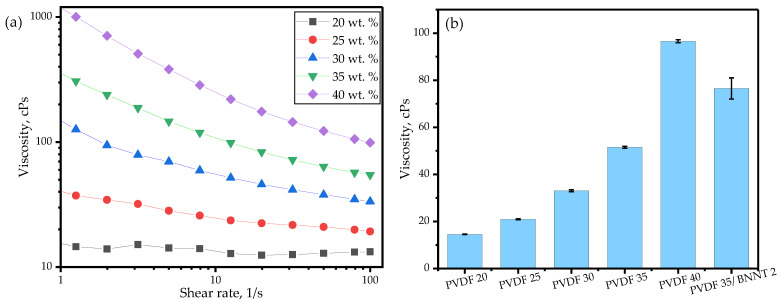
The viscosity of resin with different PVDF wt. % measured at (**a**) varying shear rate and (**b**) fixed shear rate of 100 s^−1^.

To assess viscosity, measurements were recorded over a shear rate ranging from 1–100 s^−1^ for all combinations. Figure 5a illustrates the pseudoplastic or shear-thinning flow behavior of various PVDF wt. % under a 1–100 s^−1^ shear rate sweep. As the PVDF content increased, a notable trend emerged wherein the shear thinning behavior became more pronounced. The samples with 20 wt. % PVDF exhibited Newtonian behavior, with viscosity independent of the applied shear rate, whereas higher PVDF wt. % combinations showed a drastic decrease in viscosity as the shear rate increased. This shear-thinning behavior was advantageous during the printing process as the viscosity directly correlated with flowability and self-leveling, crucial properties when evaluating the developed resins.

To compare viscosities as the PVDF wt. % increased, measurements were recorded at a shear rate of 100 s^−1^ to simulate shear loading on geometries with small and complex features. Figure 5b depicts the resulting viscosities, clearly illustrating an exponential increase in viscosity as the PVDF wt. % rose. Even though the sample with higher PVDF content exhibited a high polar phase, poor endurance was demonstrated, leading to increased instances of building platform detachment and warping over time.

Introducing BNNT fillers into the polymer matrix resulted in a 49% increase in viscosity compared to the polymer resin with 35 wt. % PVDF, as shown in Figure 5b. Nevertheless, the resin with nanofillers exhibited lower viscosity than the resin with 40 wt. % PVDF and remained within the printable range while enhancing overall piezoelectric performance, as discussed in the following sections.

### 3.4. SEM Analysis

**Figure 6:**
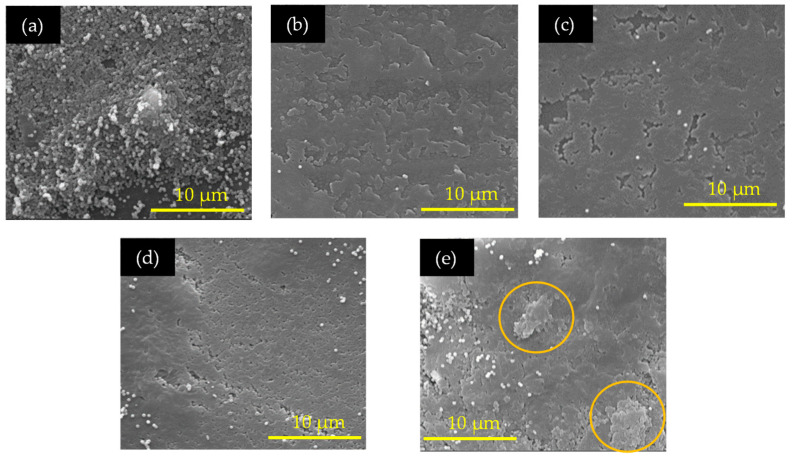
SEM images of resins with different PVDF wt. %: (**a**) 20, (**b**) 25, (**c**) 30, (**d**) 35, and (**e**) 40. The circular marker highlights the reunion phenomenon in 40 wt. % sample.

The surface morphology and homogeneity of additives with the PVDF polymer were investigated by examining 3D printed structures with varying PVDF contents (20–40 wt. %) using a FEI Quanta 650 scanning electron microscope (SEM). SEM images were captured at 20 kV, as shown in Figure 6a–e, revealing the surface quality of the printed samples, with an observed increase in polymer content mixed with other added constituents. Specifically, a combination with 40 wt. % PVDF exhibited a reunion phenomenon (highlighted with a circular marker in Figure 6e) and increased warping tendency after printing, leading to easy delamination between layers and compromising structural integrity. It was noted that a maximum range of 35 wt. % PVDF demonstrated beneficial printability and mechanical strength. Polymer softening behavior while printing samples beyond 35 wt. % was also observed in the material’s modulus, as discussed in Section 3.6.

### 3.5. Printability of Developed Polymer Resins

**Figure 7:**
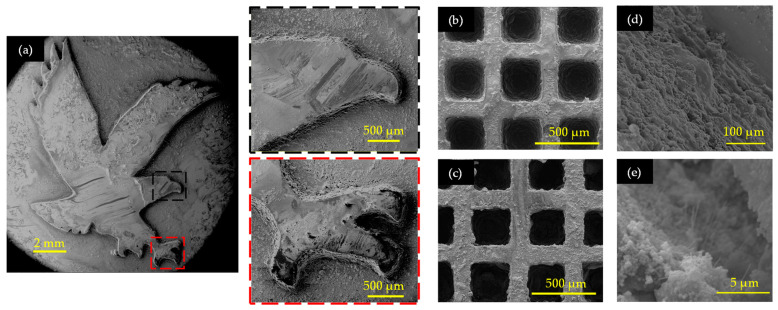
Printability of developed resins at micro-scale levels: (**a**) eagle structure with zoomed-in images of beak and claws, lattice structures with 200 µm width printed using (**b**) 35 wt. % PVDF polymer resin and (**c**) PVDF/BNNTs nanocomposite resin, (**d**) zoomed-in layer-by-layer printed nanocomposite structure, and (**e**) zoomed-in picture of BNNT nanofillers.

The developed resins demonstrated compatibility with UV-based AM techniques, facilitating the fabrication of high-resolution, micro-scale structures with complex shapes. Samples made of the developed polymer and nanocomposite resins were printed using an LCD printer for resolution inspection. Figure 7a shows a printed eagle symbol, showcasing high resolution and shape fidelity, particularly evident in intricate features such as the beak and claws, as shown in the zoomed-in images. However, the powder-based polymer resin resulted in a rough surface, necessitating refinement in the cleaning procedure before utilization. The residues on the surface, formed over time after solvent evaporation, were quickly rinsed with deionized water (DI) and blown dry with a handheld blower. Furthermore, the LCD printer proved beneficial not only for printing macro-scale structures but also for producing micro-scale structures with high resolution. SEM inspections of micro-scale scaffold lattice structures with dimensions of 200 µm width and 1 mm thickness (50 µm thick each layer) were conducted for both polymer and nanocomposite resin materials, as shown in Figure 7b,c. Additionally, the arithmetic mean height (S_a_) of the 3D printed samples was measured using the Filmetrics Profilm3D profilometer to obtain the surface roughness parameter. The surface of the nanocomposite was rough (S_a_ = 10.54 ± 0.68 µm) and flexible compared to the polymer sample (S_a_ = 7.68 ± 0.60 µm) due to the presence of BNNTs. This inspection confirmed the compatibility of the developed resin for UV-based 3D printing with better stacked layers, offering a scalable approach to manufacturing nanocomposite sensors with high resolution and structural integrity.

### 3.6. Effect of Nanofillers in PVDF Polymer’s Mechanical Property

**Figure 8:**
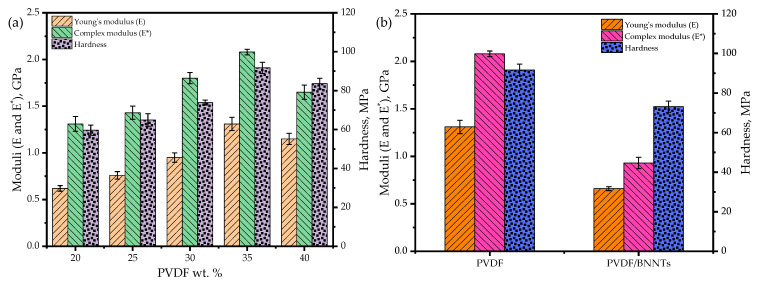
Nanoindenter results demonstrating Young’s and complex moduli for resins with (**a**) different PVDF wt. % and (**b**) added BNNT nanofillers.

To examine the modulus of the 3D printed polymer-based materials, nanoindenter analysis was employed to conduct single indentations and study the viscoelastic response, represented by the reduced modulus (*E_r_*) and the complex modulus (E*, a vector sum of storage and loss modulus), respectively. The measured *E_r_* was converted into Young’s modulus based on the Equation (3):

(3) $$\frac{1}{E_{r}}=\frac{1-\nu^{2}}{E}+\frac{1-\nu_{i}^{2}}{E_{i}}$$

where *E* and ν are the Young’s modulus and Poisson’s ratio of the material being indented (assumed polymer’s ν = 0.3) and *E_i_* and $\nu_{i}$ are the elastic modulus and Poisson’s ratio of the diamond indenter tip (*E_i_* = 1.14 GPa and $\nu_{i}$= 0.07). Figure 8a illustrates the measured (E*) and calculated (*E*) moduli alongside indentation hardness values for PVDF combinations ranging from 20 to 40 wt. %.

Based on the measured values, the modulus tended to increase with an increase in polymer content, and at 40 wt. %, the modulus dropped due to the flexible and reunion behavior of the polymer combination, as depicted in Figure 8a. A similar trend was noticed with the addition of BNNTs to the polymer, as shown in Figure 8b. This phenomenon could be ascribed to the selected UV curing process and loss of crystals, contrary to the increase in modulus observed in casting processes involving temperature and stretching [45]. Despite the low Young’s modulus with BNNT addition, the printed nanocomposite exhibited sturdiness and compliance, suitable for conformal structures, as noted in Section 3.5 regarding printability. Moreover, adding beyond 2 wt. % of BNNTs would result in agglomeration to a certain extent and hinder the adhesion between nanofillers and additives, compromising the structural integrity of the 3D printed sample [45,46].

### 3.7. Piezoelectric Property Enhancement with Added Nanofillers

Material property measurements, such as *d_33_*, *ε_r_*, and *g_33_*, play a pivotal role in assessing the performance of piezoelectric sensors. The *d_33_* value, indicative of the piezoelectric charge coefficient within the crystal structure of both the polymer and the nanocomposite, quantifies the charge generated per unit force applied in the thickness direction. Maximizing the piezoelectric response of PVDF polymer occurs when it exhibited a higher *β* phase, facilitating the alignment of the polar group along the polymer chains. The incorporation of BNNTs serve as a nucleation site for polymer crystallization, resulting in the generation of coupled electric dipoles in response to deformation, promoting stress transfer efficiency and enhancing polarization, thus strengthening the piezoelectric response.

The alignment of dipoles in the developed polymer and nanocomposite substrates, crucial for activating piezoelectricity, was achieved through polarization. Utilizing corona poling, a non-contact polarization technique involving the application of a high voltage of 8 kV for 30 min facilitated this alignment process. The measured *d_33_* values with varying PVDF contents in the developed resins post-polarization can be seen in Table 5. It was evident that the property demonstrated an increase alongside the rise in crystalline content, mirroring the trend observed in the polar phase discussed in Section 3.1.

Analysis of the measured and calculated piezoelectric properties at 100 Hz, as presented in Table 6, revealed a maximum yield of 12.20 pC/N for *d_33_* and 114.06 mVm/N for *g_33_* upon filler addition to the base polymer. However, it is noteworthy that the achieved *d_33_* value was lower than the 16–20 pC/N typically reported in the literature [47,48]. The difference can be attributed to the current composite containing only 35 wt. % polymer and 2 wt. % nanofiller, while the remaining additives lacked piezoelectric properties. This tradeoff between piezoelectric properties and printability enabled the fabrication of functional sensors through UV-based 3D printing. 

Furthermore, dielectric constant measurements at various frequencies demonstrated a 42.62% increase with BNNT addition. However, a general decrease in the *ε_r_* value from 12.08 (at 100 Hz) to 8.10 (at 1 MHz) was observed with frequency increment due to the drop in the space charge polarization effect.

### 3.8. 3D Printed Sensor Output Response

**Figure 9:**
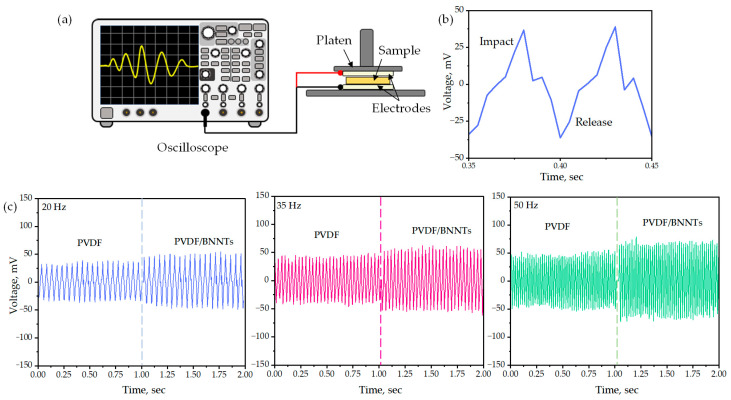
(**a**) Setup for measuring output voltage response; (**b**) peak-to-peak impact–release voltage curve. Piezoelectric response of the developed polymer and nanocomposite measured at (**c**) 20 Hz, 35 Hz, and 50 Hz.

The piezoelectric responses of 3D printed polymer and nanocomposite sensor samples, each with dimensions of 5 mm × 5 mm × 1 mm, were investigated using a platen probe and digital signal oscilloscope setup, as depicted in Figure 9a. The voltage output, indicating average peak-to-peak voltage, exhibited large positive peaks corresponding to applied stress at impact and negative values indicating damping at release, as shown in Figure 9b. Voltage responses were analyzed at 20 Hz, 35 Hz, and 50 Hz with applied longitudinal forces of 0.4 N, 0.5 N, and 0.6 N, as shown in Figure 9c. 

Table 7 summarizes the average voltage values, demonstrating the piezoelectric nature of the developed sensor material. It is evident that incorporating BNNTs into the polymer matrix showed an increase in sensor response of up to 50.13%, making it suitable for high piezoelectric response sensors.

## 4. Conclusions

This study underscores the transformative potential of AM technology in the realm of polymer nanocomposite sensors aimed to enhance piezoelectric properties and response. By investigating the structural, thermal, rheological, mechanical, and piezoelectric characteristics of novel sensor resin materials based on PVDF and a BNNT-coupled nanocomposite, this research addresses a gap in the development of UV-based, 3D printable sensor devices. Utilizing an LCD-based printer, the findings demonstrate not only the successful micro-scale printability of the nanocomposite resin but also the enhancement of its piezoelectric properties. The PVDF/BNNT nanocomposite yielded a maximum *β* fraction of 64.89%, with 12.20 pC/N (*d_33_*), 114.06 mVm/N (*g_33_*), and 12.20 (*ε_r_*). Additionally, the observed increase in piezoelectric voltage response by up to 50.13% highlights the efficacy of this approach in advancing micro-scaled sensor technology. The findings outlined in this work will provide valuable insights into the utilization of polymer-based resins in sensors and energy harvester fields. Future work will be dedicated to investigating the ferroelectric and thermal behavior at different temperatures, suitable for developing an array of embedded piezoelectric sensors in harsh environments.

## Figures and Tables

**Table 1:** Piezoelectric resin preparation compositions without and with nanofillers.

Sample	Resin, wt. %	BNNTs, wt. %
Polymer resin	100	-
Nanocomposite resin	98	2

Note: The polymer and nanocomposite resin samples contained additives such as monomers, UV absorbers, and photoinitiators.

**Table 2:** Printing parameters for polymer and nanocomposite resins using an LCD printer.

Print Parameter	Value
Layer height, mm	0.05
Bottom layer count	6
Bottom exposure time, s	55
Normal exposure time, s	30
Lift distance, mm	6
Lift speed, mm/min	60
Retract speed, mm/min	150

**Table 3:** Calculated relative polar phase fraction in developed polymer-based resins.

Sample, wt. %	F (*β*), %
PVDF 20	53.86
PVDF 25	56.08
PVDF 30	57.21
PVDF 35	60.01
PVDF 40	61.08
PVDF 35/BNNTs 2	64.89

**Table 4:** Melting and crystallinity parameters of polymer-based resin combinations obtained through DSC measurements.

Sample, wt. %	*T_m_*, °C	Δ*H_m_*, J/g	*X_c_*, %
PVDF 20	133.36	9.79	30.49
PVDF 25	133.88	12.02	37.43
PVDF 30	134.33	13.73	42.76
PVDF 35	135.57	15.20	47.34
PVDF 40	138.71	15.78	49.14
PVDF 35/BNNTs 2	157.60	7.32	23.79

**Table 5:** Measured piezoelectric strain coefficient for 3D printed resins with different PVDF contents after polarization.

PVDF Sample, wt. %	*d_33_*, pC/N
20	3.40 ± 0.15
25	4.33 ± 0.19
30	5.67 ± 0.36
35	7.34 ± 0.20
40	8.14 ± 0.17

**Table 6:** Effect of adding fillers on piezoelectric properties, measured at 100 Hz.

Sample, wt. %	*d_33_*, pC/N	*ε_r_*	*g_33_*, mVm/N
PVDF 35	7.34 ± 0.20	8.47 ± 0.01	97.85 ± 0.11
PVDF 35/BNNTs 2	12.20 ± 0.83	12.08 ± 0.02	114.06 ± 0.43

**Table 7:** Piezoelectric sensor voltage response measured at different frequencies.

Sample		Voltage, mV	
	20 Hz	35 Hz	50 Hz
PVDF 35	33.09 ± 4.43	38.58 ± 3.25	41.75 ± 4.93
PVDF 35/BNNTs 2	49.24 ± 5.17	54.23 ± 2.92	62.68 ± 6.36

## Data Availability

Data are contained within the article.
